# The application of whole-exome sequencing in the early diagnosis of rare genetic diseases in children: a study from Southeastern China

**DOI:** 10.3389/fped.2024.1448895

**Published:** 2024-10-08

**Authors:** Guihua Lai, Qiying Gu, Zhiyong Lai, Haijun Chen, Junkun Chen, Jungao Huang

**Affiliations:** Central Laboratory, Ganzhou Maternal and Child Health Hospital, Ganzhou, Jiangxi, China

**Keywords:** whole-exome sequencing, genetic diseases, genetic diagnosis, children, rare disease

## Abstract

**Background:**

Genetic diseases exhibit significant clinical and genetic diversity, leading to a complex and challenging diagnostic process. Exploiting novel approaches is imperative for the molecular diagnosis of genetic diseases. In this study, we utilized whole-exome sequencing (WES) to facilitate early diagnosis in patients suspected of genetic disorders.

**Methods:**

This retrospective analysis included 144 patients diagnosed by singleton-WES Trio-WES between January 2021 and December 2023. We investigated the relevance of diagnosis rates with age, clinical presentation, and sample type.

**Results:**

Among the 144 patients, 61 were diagnosed, yielding an overall diagnostic rate of 42.36%, with Trio-WES demonstrating a significantly higher diagnostic rate of 51.43% (36/70) compared to singleton-WES at 33.78% (25/74) (*p* < 0.05). Global developmental delay had a diagnosis rate of 67.39%, significantly higher than muscular hypotonia at 30.43% (*p* < 0.01) among different clinical phenotypic groups. Autosomal dominant disorders accounted for 70.49% (43/61) of positive cases, with autosomal abnormalities being fivefold more prevalent than sex chromosome abnormalities. Notably, sex chromosome abnormalities were more prevalent in males (80%, 8/10). Furthermore, 80.56% (29/36) of pathogenic variants were identified as *de novo* mutations through Trio-WES.

**Conclusions:**

These findings highlight the effectiveness of WES in identifying genetic variants, and elucidating the molecular basis of genetic diseases, ultimately enabling early diagnosis in affected children.

## 1 Introduction

Genetic diseases, caused by genetic alterations leading to abnormal function, result in diverse clinical phenotypes, which can occur at all ages and are often congenital. Therefore, it is particularly common in pediatric patients. With the advancement of life science and society, the spectrum of childhood diseases has undergone significant changes. According to the estimate of the World Health Organization in 2007, the current birth defect rate in China is 5.6%, significantly higher than the 4.72% in developed countries (1). Furthermore, birth defects account for 20.13% of child deaths in China (2). Therefore, the diagnosis and treatment of genetic diseases and the follow-up course management become crucial in the clinical work of pediatricians. However, children with potential genetic diseases often exhibit multiple systems, multiple organs, and diverse symptoms, which are more complex than ordinary pediatric patients and are easily misdiagnosed and missed. They also have a higher proportion of hospitalization, accounting for 9%–15%, and higher mortality rates (1.0%–1.3%) (3–5). Assessing the diagnosis of genetic diseases in children requires highly vigilant specialist physicians, often requiring complex clinical examinations and evaluations, which are time-consuming and costly. Even after detailed diagnostic evaluation, the majority of children still been diagnosed unclearly. Therefore, due to the often complex clinical manifestations of genetic disease, a comprehensive diagnostic technology is required to achieve early diagnosis. In recent years, with the continuous development of molecular diagnosis and the ability to reveal the genetic causes of genetic diseases, medical practice is undergoing a revolutionary transformation from traditional symptom-based diagnosis to modern cause-based diagnosis (6). Choosing the right molecular genetic diagnostic strategy can help shorten the diagnostic odyssey and avoid the economic burden of redundant diagnostic testing. This can help accurately intervene in diseases related to genetic causes with clearly defined locations, thereby improving disease prognosis (7). This study analyzed the clinical records and diagnostic data of 144 cases diagnosed by whole-exome sequencing (WES) to evaluate the diagnostic efficacy of WES in different ages and clinical phenotypes.

## 2 Materials and methods

### 2.1 Study design and participants

This was a retrospective cohort study conducted at the Clinical Genetics Laboratory of the Ganzhou Maternal and Child Health Hospital. Pediatric patients were recruited between January 2021, and December 2023. The inclusion criteria were as follows: (1) children with an unclear clinical diagnosis for whom genetic disorders were considered; and (2) an order for Next-Generation Sequencing and complete medical history. Notably, Patients were excluded if they were undergoing emergency surgery or external blood transfusion. Whole exome sequencing was conducted as Trio-WES (both parents and their affected child sequenced simultaneously) to effectively detect *de novo* and compound heterozygous variants or as singleton-WES (only the affected individual sequenced) when parental samples were not available. Trio-WES were performed by 70 patients of this cohort with non-consanguineous healthy parents, and the remaining 74 probands underwent singleton-WES. We collected basic information about each patient through a retrospective review of medical records and examined the correlation between clinical information and diagnostic findings. The study was approved by the ethics committee of the Ganzhou Maternal and Child Health Hospital (202396). The legal guardians of the participating children gave their signed, informed consent for their children to be included in the study.

### 2.2 Next-generation sequencing

Blood samples of patients and any participating family members were collected, and genomic DNA was extracted using the QIAamp DNA Mini Kit (Hilden, Germany) following the manufacturer's protocol. The coding exons of target genes were captured using an Exome Panel v2.0 (Nanodigmbio, Nanjing), and libraries generated from enriched DNA were sequenced using the Illumina NovaSeq 6000 platform (Illumina Inc., San Diego, CA, USA) in paired-end mode. The average on-target sequencing depth for exome sequencing was 90X, and more than 98% of target bases had a coverage of over 20X. The sequencing reads were aligned to the human reference genome (UCSC GRCh37/hg19) using the Burrows-Wheeler Aligner. Subsequently, single nucleotide variant (SNV) and small insertion/deletion (Indel) detection were conducted. Finally, variant filtering was performed with the PhenoPro (8) phenotype-scoring algorithm. Copy number variants (CNV) analysis is primarily based on sequencing depth or read counts. The analysis process includes GC correction of the samples, normalization of sample data within batches, calculation of log2Ratio and Z-scores, and finally, hierarchical clustering analysis and segmentation process, resulting in a VCF file containing CNV information. The causative variants detected through Trio/singleton-WES were subsequently confirmed by PCR or Sanger sequencing in the proband and parents if available using a 3500XL Genetic Analyzer (Applied Biosystems) according to the manufacturer's specifications. The variant's pathogenicity was determined using the criteria established by the American College of Medical Genetics and Genomics (9).

### 2.3 Statistical analysis

The significance of the diagnostic rate of Trio-WES vs. singleton-WES (*p* values) was calculated by one-tailed Fisher's exact test. All other comparisons were done by a two-tailed Fisher's exact test. A p value of 0.05 was used as a significance threshold. The statistical analyses were conducted using the software package SPSS (version 26.0, IBM Corp., 2019).

## 3 Results

### 3.1 Demographics of clinical feature

From January 1, 2021, to December 31, 2023, a total of 144 unrelated patients were included in the study. The cohort consisted of 85 males and 59 females, with a median age of 4 years (range 0–17). All participants underwent WES for suspected genetic disorders. Among the 144 pediatric cases, there were 100 young children (aged 5 and under). Notably, 55% (55 out of 100) of the young children underwent Trio-WES analysis. In contrast, only 15 out of 44 older children (over 5 years old) underwent this analysis, representing 34.09% (*p* < 0.05). The clinical manifestations observed in the patients included global developmental delay (*n* = 46, 31.94%), intellectual disability (*n* = 44, 30.56%), abnormal metabolism (*n* = 26, 18.06%), dysmorphic facial features (*n* = 25, 17.36%), seizures (*n* = 24, 16.67%), muscular hypotonia (*n* = 23, 15.97%), and autistic behavior (*n* = 19, 13.19%), as detailed in Table 1, Supplementary Table S1 and Supplementary Table S2.

**Table 1:** Molecular diagnostic rates of WES by assay type, age and clinical phenotype.

Characteristics	All patients (*N* = 144)	Diagnostic variants P + LP (*N* = 61)	Diagnostic rate (%)
Sex, *n* (%)			
Male	85 (59.03)	34 (55.74)	40
Female	59 (40.97)	27 (44.26)	45.76
Age, *n* (%)			
≤ 5 y	100 (69.44)	48 (79.69)	48
> 5 y	44 (30.56)	13 (21.31)	29.55
Type of samples, *n* (%)			
Singleton-WES	74 (51.39)	25 (40.98)	33.78
Trio-WES	70 (48.61)	36 (59.02)	51.43
Major symptoms, *n* (%)			
Global developmental delay (HP:0001263)	46 (31.94)	31 (50.82)	67.39
Intellectual disability (HP:0001249)	44 (30.56)	28 (45.90)	63.64
Abnormal metabolism (HP:0032245)	26 (18.06)	7 (11.48)	26.92
Dysmorphic facial features (HP:0001999)	25 (17.36)	9 (14.75)	36
Seizures (HP:0001250)	24 (16.67)	12 (19.67)	50
Muscular hypotonia (HP:0001252)	23 (15.97)	7 (11.48)	30.43
Autistic behavior (HP:0000729)	19 (13.19)	8(13.11)	42.11

P, pathogenic; LP, Likely pathogenic. A patient may have dual or even more phenotypes in our cohort.

### 3.2 Diagnostic yield of WES

The diagnostic results were obtained in 61 out of 144 patients and the overall diagnosis rate of WES was 42.36%, with a significantly higher diagnosis rate of 51.43% (36/70) in Trio-WES than 33.78% (25/74) in singleton-WES (*p* < 0.05). Furthermore, we grouped children according to age at different stages and found that the diagnosis rate of young children was significantly higher at 48% (48/100) than that of older children at 29.55% (13/44, *p* < 0.05). To explore the diagnostic rate of WES in terms of clinical phenotype, we analyzed the molecular diagnostic rate of clinical phenotype subgroups, as shown in Table 1. We found that the diagnostic rate in the global developmental delay patient group was (67.39%, 31/46), which was significantly higher than the diagnostic rate in the muscular hypotonia patient group (30.43%, 7/23, *p* < 0.01). Moreover, the diagnosis rate in the intellectual disability patient group was (63.64%, 28/44), which was significantly higher than the diagnosis rate in the abnormal metabolism patient group (26.92%, 7/26, *p* < 0.01). These findings suggested significant differences in diagnostic rates across clinical phenotypes.

### 3.3 Analysis of genetic variation

**Figure 1:**
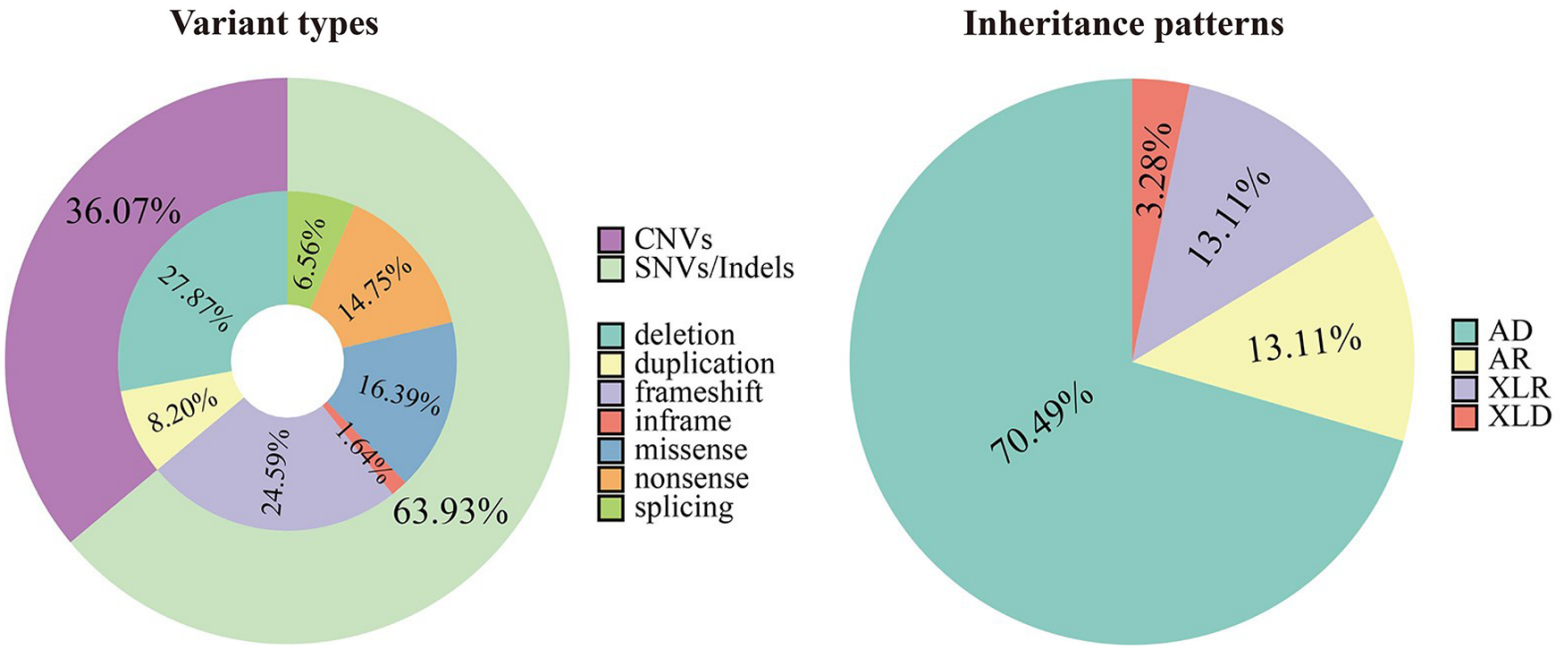
Variant types and inheritance patterns in 61 children with genetic diseases.

**Figure 2:**
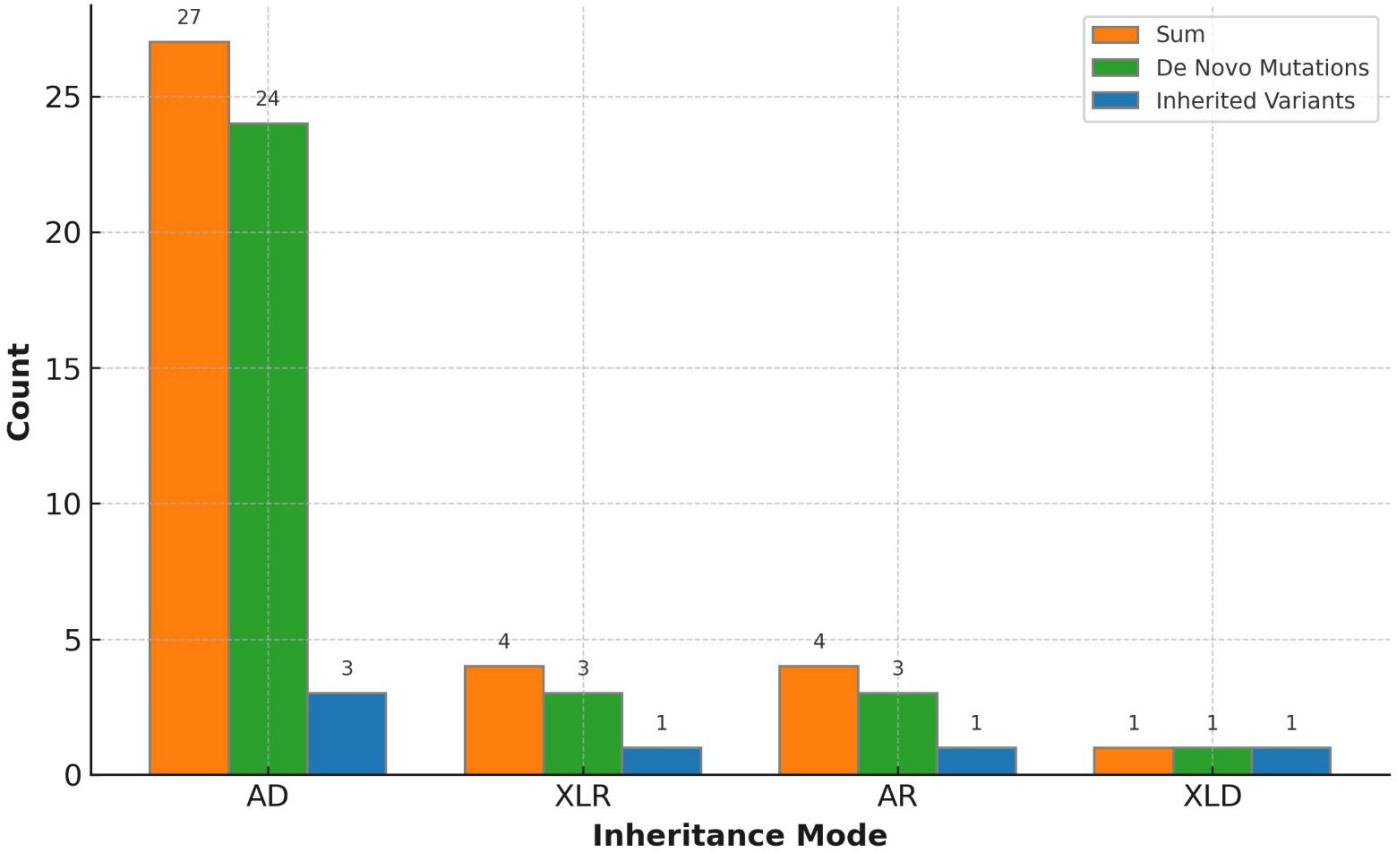
Variant inheritance of the 36 positive trio-WES cases.

An overall diagnostic yield of 42.36% (61/144) was obtained through the combined analysis of Indels, CNVs, and SNVs. This included 63.93% (39/61) from SNV/Indel analysis and 36.06% (22/61) from exome-based CNV analysis. CNV analysis identified 22 pathogenic CNVs, with 27.87% (17/61) being deletions and 8.2% (5/61) duplications, ranging from 406 Kb to 25.3 Mb. SNV/Indel analysis identified 39 pathogenic or likely pathogenic variants in 33 different genes, based on ACMG guidelines. Among these, 24.59% (15/61) were frameshift variants, 16.39% (10/61) were missense variants, 14.75% (9/61) were nonsense variants, 6.56% (4/61) were splice variants, and 1.64% (1/61) were inframe variant (Figure 1). Specifically, among these positive patients, 70.49% (43/61) had autosomal dominant (AD) disorders, with global developmental delay (60.47%, 26/43) and intellectual disability (53.49%, 23/43) as the most common clinical phenotypes. Furthermore, 13.12% (8/61) had autosomal recessive (AR) disorders, with abnormal metabolism (87.5%, 7/8) being the most common clinical phenotype. Additionally, 13.12% (8/61) had X-linked recessive (XLR) disorders, which were exclusively in males, with muscular hypotonia (37.5%, 3/8) and intellectual disability (37.5%, 3/8) being the most common clinical phenotype. In 3.27% (2/61) of the children with X-linked dominant (XLD) disease, all of them were females, and the clinical phenotype of the two children was characterized primarily by intellectual disability. In the 61 positive children, the prevalence of autosomal abnormalities was above fivefold higher than sex chromosome abnormalities (51/10). Sex chromosome abnormalities were predominantly found in 80% (8/10) of males (Table 2). Furthermore, 80.56% (29/36) of pathogenic variants were identified as *de novo* mutations by Trio-WES, and the majority of these children had AD conditions 82.76% (24/29), while AR conditions was found in only 3.45% (1/17, Figure 2). The most common clinical phenotypes in patients with *de novo* mutations were intellectual disability (55.17%, 16/29), global developmental delay (44.83%, 13/29), and seizures (24.14%, 7/29).

**Table 2:** Inheritance patterns of different clinical phenotype subgroups.

Characteristics	Inheritance			
	AD	AR	XLD	XLR
Sex, *n* (%)				
Male	22	4	0	8
Female	21	4	2	0
Major symptoms, *n* (%)				
Global developmental delay (HP:0001263)	26	2	1	2
Intellectual disability (HP:0001249)	23	0	2	3
Seizures (HP:0001250)	11	0	0	2
Dysmorphic facial features (HP:0001999)	8	0	0	1
Autistic behavior (HP:0000729)	7	0	0	0
Muscular hypotonia (HP:0001252)	3	0	1	3
Abnormal metabolism HP:0032245	0	7	0	0

A patient may have dual or even more phenotypes in our cohort.

## 4 Discussion

Genetic disorders, categorized as chromosomal, monogenic, polygenic, and mitochondrial disorders, involve congenital malformations, physiological defects, or metabolic abnormalities. The incidence of genetic disorders is estimated to be between 40 and 82 per 1,000 live births (10). For most patients with genetic disorders, a reliable diagnosis is essential to improve management and quality of life for patients and their families. Some studies have shown that molecular diagnosis of genetic diseases is an important and indispensable method for their accurate diagnosis (11). Genome sequencing technology has been shown to be an important tool for the discovery of new disease-associated genetic loci and it is an effective alternative to gene panel testing (12, 13). It is known that exonic regions comprise only about 1% of the entire human genome but contain 85% of the disease-causing variants (14). In this study, we used WES to directly detect the causative genes of genetic diseases for early diagnosis of diseases. Our results showed that 42.36 percent (61/144) of patients received a molecular diagnosis. Although there is no cure for most genetic disorders, symptomatic treatment of patients with a clear molecular etiology can significantly reduce the burden of events and improve subsequent clinical management (15). Furthermore, in our study, 79% of the patients with genetic disorders were young children, and our results are consistent with the early onset of genetic disorders and the fact that they are mostly congenital. Furthermore, among 144 children suspected of having genetic diseases, we found a higher percentage of male than female patients, which we considered to be due to the presence of sex chromosome variants. Our results showed that there are far more males than females with X-linked genetic disorders in the population, and the results could be more significant if the sample size is increased.

Furthermore, the results indicated that the highest percentage of patients were associated with AD conditions (70.49%). Using Trio-WES, we found that the variants in these patients were predominantly *de novo* mutations. It is possible due to the fact that AD conditions have a certain rate of disability and mortality, most families reject having children or undergo prenatal diagnosis (16, 17), and thus do not have a similar family history. Our study found that the patients associated with AR conditions accounted for 13.12%, and the disease phenotype was predominantly characterized by aberrant metabolism. Most variants associated with AR conditions were inherited from parents, with a higher prevalence observed in consanguineous marriage families (18). In this study, no Y-linked genetic diseases were detected.

Previous studies have shown that the diagnostic sensitivity of WES varies depending on the organ system involved (19). In this study, we grouped 144 patients with major clinical phenotypes and found significant differences in diagnostic rates between phenotype groups. Global developmental delay had the highest positive rate of 67.39%, followed by Intellectual disability (63.64%). It has been reported that the diagnostic rate of WES in children with neurological abnormalities was significantly higher than that of non-neurological abnormalities (20, 21). In our study, the diagnostic rates of global developmental delay, intellectual disability, autistic behavior, and seizures were higher than dysmorphic facial features, muscular hypotonia, and abnormal metabolism, which is consistent with the trend of previous findings (20). The low diagnostic rate of non-neurologic abnormalities suggested that the proportion of unknown genes, complex structural variation, or non-genetic underlying mechanisms may be greater. Compared to WES, Whole genome sequencing (WGS), with its superior and comprehensive genome coverage, enhances the detection of deep intronic regions and complex structural variants. However, considering efficacy and difficulty of interpretation, WES is more applicable for clinical testing currently. In recent years, as the cost of WGS decreases and the interpretation level of genetic testing improves, future studies could be considered to employ this technique on undiagnosed patients but highly suspected with genetic diseases to discover novel genes or pathogenic variants (22, 23). In addition, we found that the diagnostic rate of Trio-WES was significantly higher than that of singleton-WES. Trio-WES can determine whether the variants detected in proband samples are inherited from the parents, which is extremely essential in analyzing *de novo* mutations. Previous studies have found that patients with intellectual disability (24), global developmental delay (25), and epilepsy (26) are the most common groups affected by *de novo* mutations. Our results showed that *de novo* mutations were present in up to 80% (29/36) of all pathogenic variants, with the most common clinical phenotype being Intellectual disability 55.17% (16/29), followed by global developmental delay 44.83% (13/29). We found that the percentage of *de novo* pathogenic variants inherited as AR was only 3.45% (1/29), which may be due to the fact that *de novo* mutations are limited by the frequency of mutations in the species (27). In this study, the higher detection rate of *de novo* mutations was attributed to the Trio-WES testing strategy, which enhanced the clinical interpretation of the pathogenicity of the variants through co-testing of the proband and its parental samples (28). Therefore, the testing protocol of Trio-WES should be prioritized to improve the diagnostic rate when parents are available (29). However, there are limitations of using the WES approach, as WES targets only the coding region of the genome and may not reliably detect CNVs at the single-gene or exon level, which means WES may not accurately detect non-coding, deep intronic regions, or copy number changes, as well as complex genomic structural variants, such as gene rearrangement or inversion. In addition, variant of uncertain significance (VUS) results were detected and reported. Incorrect interpretations of these variants can cause patient anxiety and lead to inappropriate patient management.

In recent years, with the improvement of tools for classifying genetic variants and the continuous updating of genetic databases and clinical phenotypes (30), reanalysis of WES may improve the diagnosis of the disease (13, 31). Therefore, enhanced follow-up and reanalysis of patients with non-diagnostic findings is important for our subsequent studies. In addition, likely pathogenic variants only mean 90% chance of pathogenicity and may not be diagnostic. WES is a phenotype-driven analysis, providing accurate clinical phenotypes is essential for the lab to filter out the potential diagnostic findings. Therefore, the combination of clinical phenotype with genetic finding to make a clinical judgment is crucial in the diagnosis.

## 5 Conclusions

In this study, 144 patients suspected of genetic diseases were analyzed by WES, which revealed significant differences in diagnostic efficiency among different clinical phenotypes. Besides, we found that AD disorders dominated by *de novo* mutations were the most prevalent, variants associated with AR and XLR were inherited from parents. In conclusion, we proposed that Trio-WES can efficiently identify the genetic causes of the diseases, and improve the diagnostic rate of the genetic diseases in clinical work.

## Data Availability

The original data is available online (https://figshare.com/s/431c345a768b15dee77a). Requests to access the raw data should be directed to the corresponding author.

## Supplementary material

Supplementary Table S1 Detailed information on each case.

Supplementary Table S2 Numbers of cases with each symptom.
